# Cost-effectiveness of adding novel or group 5 interventions to a background regimen for the treatment of multidrug-resistant tuberculosis in Germany

**DOI:** 10.1186/s12913-017-2118-2

**Published:** 2017-03-08

**Authors:** Daniel Wirth, Ramesh Dass, Robert Hettle

**Affiliations:** 1 Health Economics & Market Access, Janssen-Cilag GmbH, Johnson & Johnson Platz 1, 41470 Neuss, Germany; 2 Janssen-Cilag Ltd, High Wycombe, UK; 3 PAREXEL International, London, UK

**Keywords:** Cost-effectiveness, Multidrug-resistant tuberculosis, Bedaquiline, Delamanid, Linezolid, QALY, LYG, ICER

## Abstract

**Background:**

Treatment of multidrug-resistant tuberculosis (MDR-TB) is complex, lengthy, and involves a minimum of four drugs termed a background regimen (BR), that have not previously been prescribed or that have proven susceptible to patient sputum culture isolates. In recent years, promising new treatment options have emerged as add-on therapies to a BR. The aim of this study was to evaluate the long-term costs and effectiveness of adding the novel or group 5 interventions bedaquiline, delamanid, and linezolid to a background regimen (BR) of drugs for the treatment of adult patients with pulmonary multidrug-resistant tuberculosis (MDR-TB), within their marketing authorisations, from a German healthcare cost-effectiveness perspective.

**Methods:**

A cohort-based Markov model was developed to simulate the incremental cost-effectiveness ratio of bedaquiline plus BR, delamanid plus BR, or linezolid plus BR versus BR alone in the treatment of MDR-TB, over a 10-year time horizon. Effectiveness of treatment was evaluated in Quality-Adjusted Life-Years (QALYs) and Life-Years Gained (LYG), using inputs from clinical trials for bedaquiline and delamanid and from a German observational study for linezolid. Cost data were obtained from German Drug Directory costs (€/2015), published literature, and expert opinion. A 3% yearly discount rate was applied. Probabilistic and deterministic sensitivity analyses were conducted.

**Results:**

The total discounted costs per-patient were €85,575 for bedaquiline plus BR, €81,079 for delamanid plus BR, and €80,460 for linezolid plus BR, compared with a cost of €60,962 for BR alone. The total discounted QALYs per-patient were 5.95 for bedaquiline plus BR, 5.36 for delamanid plus BR, and 3.91 for linezolid plus BR, compared with 3.68 for BR alone. All interventions were therefore associated with higher QALYs and higher costs than BR alone, with incremental costs per QALY gained of €22,238 for bedaquiline, €38,703 for delamanid, and €87,484 for linezolid, versus BR alone. In a fully incremental analysis, bedaquiline plus BR was the most cost-effective treatment option at thresholds greater than €22,000 per QALY gained. In probabilistic analyses, the probability that bedaquiline plus BR was the most cost-effective treatment strategy at a willingness-to-pay threshold of €30,000 was 54.5%, compared with 22.9% for BR alone, 18.2% for delamanid plus BR, and 4.4% for linezolid.

**Conclusions:**

In Germany, the addition of bedaquiline, delamanid, or linezolid to a BR would result in QALY gains over BR alone. Based on this analysis, bedaquiline is likely to be the most cost-effective intervention for the treatment of MDR-TB, when added to a BR regimen at thresholds greater than €22,000 per QALY.

**Electronic supplementary material:**

The online version of this article (doi:10.1186/s12913-017-2118-2) contains supplementary material, which is available to authorized users.

## Background

Multidrug-resistant tuberculosis (MDR-TB) is a form of tuberculosis (TB) that is resistant to at least the two most effective first-line therapeutic drugs, isoniazid and rifampicin [1]. MDR-TB is a persistent, and in some regions, increasing public health concern: the incidence of MDR-TB in Europe in 2013 was 16.9% among new TB cases and 48.0% among previously treated cases [2].

In Germany, the total number of MDR-TB cases has increased year-on-year between 2010 and 2013, reaching 100 to 102 reported cases in 2013, depending on the reporting method used [2, 3]. Further, three cases of extensively drug-resistant (XDR)-TB in Germany were reported in 2013 [3]. These statistics reflect a trend towards increasing antibiotic resistance that is mirrored throughout many regions of Europe, both in tuberculosis and in other infectious diseases [4].

Treatment of MDR-TB is complex and involves a minimum of four drugs that have not previously been prescribed or that have proven susceptible to patient isolates (termed a background regimen, or BR). The recommended total duration of treatment is 18–24 months and patient isolation is recommended until sputum culture conversion is achieved [5–8].

Despite these lengthy and resource-intensive regimens, success rates are suboptimal; the European Centre for Disease Control (ECDC) estimated that just 38% of patients who started MDR-TB treatment in 2011 were cured 24 months later. 235 of 1386 patients starting MDR-TB treatment died, representing a 17% mortality rate [2, 9]. For Germany, the Robert Koch Institute published very recent data with similar treatment success rates (34%) [10]. Further, many MDR-TB therapies are highly toxic and are associated with side effects including nausea, vomiting, peripheral neuropathy, nephrotoxicity, haemotoxicity, and ototoxicity. These side effects contribute to an additional treatment and monitoring burden, negatively affect patient quality of life, and decrease patient adherence, therefore affecting the probability of treatment success [6]. All of these factors contribute to the high economic burden of MDR-TB; a recent study estimated that the direct costs (in 2012€) of MDR-TB treatment in Germany totalled €64,429 per patient, with a further €17,722 to €44,304 in lost productivity costs [11].

It is therefore necessary to introduce more effective MDR-TB treatment regimens that lead to improved patient outcomes and reduced disease transmission, while also demonstrating a manageable adverse event (AE) profile. Recently, three promising treatment options have emerged as add-on therapies to a BR: bedaquiline, linezolid, and delamanid. These are novel or Group 5 interventions that have been shown to improve efficacy outcomes in patients with MDR-TB over BR alone [12–14]. Bedaquiline and delamanid are novel interventions, while linezolid has been repurposed as an off-label treatment option in MDR-TB.

However, new healthcare interventions must be evaluated in the context of fixed healthcare budgets. In order to effectively allocate limited healthcare resources, it is important to establish not only the efficacy, but also the cost-effectiveness of new interventions for MDR-TB. Bedaquiline plus BR has previously been demonstrated to be cost-effective versus BR alone in a range of low-income, middle-income, and high-income settings including Germany [15–17]. Delamanid has also been shown to be cost-effective versus BR in the German setting [18]. However, no published study to date has evaluated the cost-effectiveness of these three interventions within the same model structure, or evaluated the cost implications of associated AEs.

The aim of this study was to evaluate the cost-effectiveness of adding bedaquiline, delamanid, or linezolid to a BR of drugs in the German setting, including the economic impact of AE management for each intervention.

## Methods

### Model overview

A cohort-based Markov state transition model was developed to evaluate the long-term costs and effectiveness of adding bedaquiline, delamanid, or linezolid to a BR, compared with BR alone in the treatment of adult patients with pulmonary MDR-TB. The model was originally developed for the UK healthcare system [17], and subsequently adjusted to the perspective of the German Statutory Health Insurance system [16].

A cohort of 100 patients with MDR-TB was included in the model simulation, reflecting current epidemiological data for Germany at the time of model design [2]. Of these patients, 87.7% were assumed to be treated in the inpatient setting, over a mean duration of 89.1 days [11].

Outcomes considered in the model included direct costs, quality-adjusted life-years (QALY), life-years gained (LYG), and incremental cost-effectiveness ratio (ICER). Both costs and effectiveness were discounted in the base case at an annual rate of 3% following local guidelines [19]. A 1-month cycle length and half-cycle correction were applied to estimate costs and outcomes, with a 10 year time horizon.

### Model structure

**Fig. 1:**
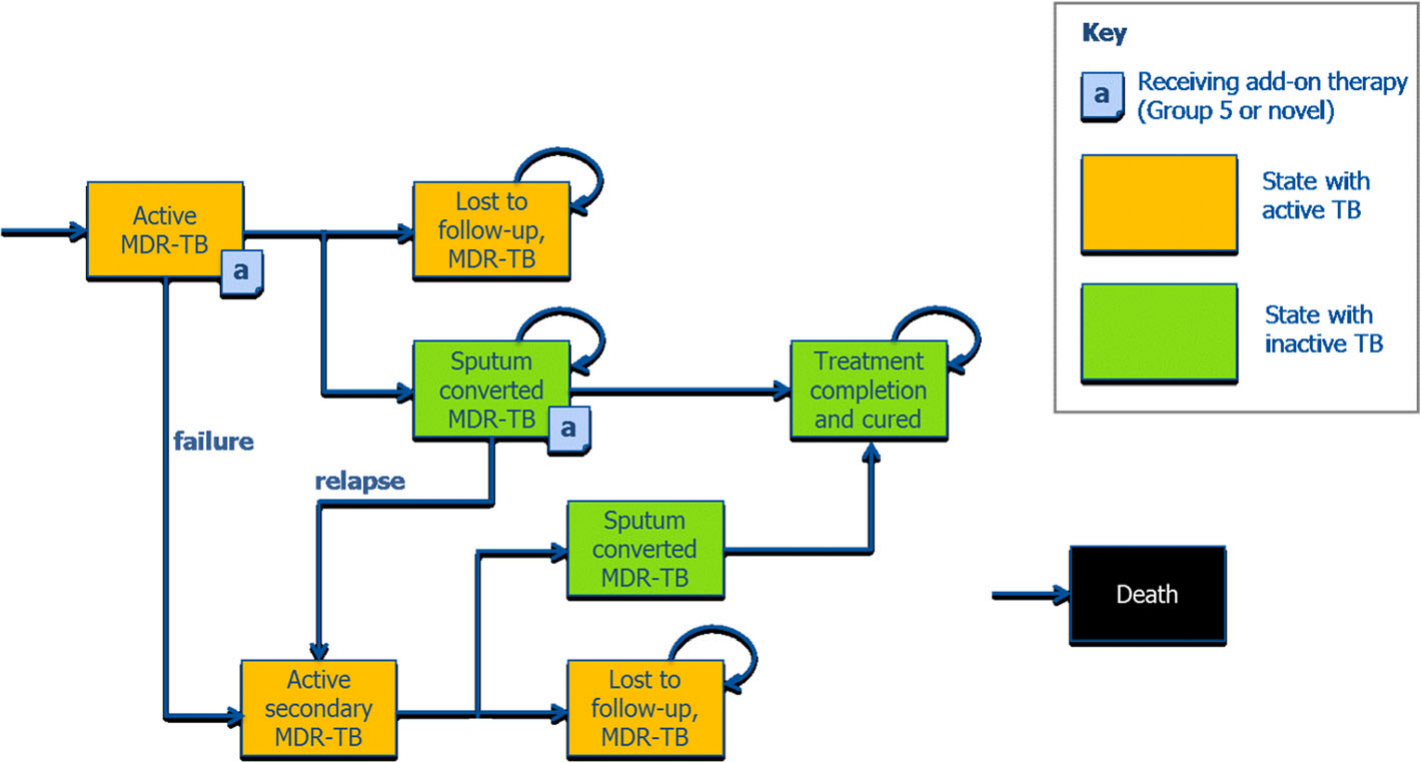
Model structure, adopted from [16, 17]. Transitions to the “Death” state are possible from every state, but not shown on the diagram for better clarity. MDR-TB: Multidrug resistant tuberculosis; TB: tuberculosis

The model structure comprised six health states: active MDR-TB, sputum culture converted MDR-TB, treatment completion (representing cure), surgery, lost to follow-up (permanent treatment failure), and death (Fig. 1) [17]. The goal of drug treatment was to induce and maintain sputum culture conversion until treatment completion (assumed equivalent to MDR-TB cure) [20]. Patients failing to achieve sputum culture conversion during the first year of the simulation were considered treatment failures and transitioned to the ‘active secondary MDR-TB’ state at month 12 to begin a new treatment course. Patients who failed to achieve culture conversion following the new treatment course were assumed to occupy the ‘active secondary MDR-TB’ state until death or loss to follow-up, for the purpose of model simplification.

The probabilities of intervention, loss to follow-up, and death were based on the literature [14, 21–24]. A summary of the transition probabilities applied in the model is shown in Table 1.

**Table 1:** Transition probabilities in the model

Health state	Transition to	Monthly probability (%)	Distribution for probabilistic analysis	Source
Active TB (MDR-TB; secondary MDR-TB and XDR-TB)	Sputum culture converted	Variable	Multivariate log-normal distribution	[14]
	Lost to follow-up	0.39	Beta	[14]
	Death	MDR-TB, no cure: 2.21, MDR-TB, cured: 0.32 XDR-TB, no cure: 2.69% XDR-TB, cured: 0.39	Beta	[21, 22]
Lost to follow-up	Death	6.87	Beta	[24]
Sputum culture converted	Active secondary MDR-TB (relapse)	0.98	Beta	[14]
	MDR-TB or active XDR-TB (relapse)	(21.4 to XDR-TB; 78.6 to MDR-TB)		[14]
Treatment completion and cured	Active secondary MDR-TB (reoccurrence)	0.20	Beta	[21]
	MDR-TB or active XDR-TB (reoccurrence)	(21.4 to XDR-TB; 78.6 to MDR-TB)		[14]

*MDR* multidrug-resistant, *TB* tuberculosis, *XDR* extensively drug-resistant

### Efficacy and quality of life inputs

Efficacy data for the interventions under consideration were sourced from the results of Phase 2, placebo-controlled clinical trials for bedaquiline [14] and delamanid [12]. In both studies, the effect of treatment was evaluated in terms of the hazard ratio for time to sputum culture conversion (SCC), with bedaquiline and delamanid added to BR being associated with statistically significant improvements in the rate of SCC versus BR alone.

The rate of SCC in the population treated with add-on therapies was calculated by multiplying the hazard rate of culture conversion in the BR alone population by the hazard ratio of sputum culture conversion for treatment plus BR, versus BR alone. This calculation generated a treatment-related hazard rate, which was subsequently used to estimate the probability of culture conversion while receiving add-on therapies (Table 2). The bedaquiline trial publication reported the hazard ratio for SCC versus placebo as 2.44 (95% confidence interval [CI]: 1.57–3.80) [14].

**Table 2:** Summary of relative efficacy of adding novel or group 5 interventions to a BR

Intervention	Time to SCC	Mean treatment effect as Relative Risk RR (SE)	Discontinuation rate per month (%)	Source/assumption
Bedaquiline vs. placebo	83 days vs. 125 days	2.44 (0.57)	0.87%	[14]
Delamanid vs. placebo	Not reported (HR of 0.58 reported only for placebo vs. delamanid)	1.73 (0.38)	1.26%	Analysis of data reported in [12]
Linezolid vs. placebo	103 days vs. 65 days	1.28 (0.57)	3.1%	[13] see main text

*BR* Background regimen, *RR* relative risk, *SE* Standard Error, *SCC* sputum culture conversion

For delamanid, comparative efficacy versus placebo was derived using a Bayesian meta-analysis. For the meta-analysis, a fixed effects model was fitted to aggregated summary data from the study by Gler and colleagues [12]. The Bayesian model consisted of a binomial likelihood function, complementary log-log link function, and vague prior distributions. The estimated hazard ratio for delamanid versus placebo was 1.73 (95% CI: 1.153–2.627), based on a calculation of 1 divided by 0.58 (the reported hazard ratio for increased time to SCC with placebo vs. delamanid) [12]. Data for delamanid from Gler 2012 were only available for 8 weeks but in the model, the duration of add-on therapy for delamanid was assumed to be 24 weeks. In the absence of additional data, it was conservatively assumed that the hazard ratio for SCC at 8 weeks was maintained for 24 weeks.

Linezolid is used in an off-label setting for MDR-TB; data from a German observational study were used to determine relative efficacy for linezolid [13] (Table 2). In this study, patients were treated according to WHO recommendations, using fluoroquinolones, injectable agents and other second-line oral agents (BR), linezolid being one agent in this regimen. Patients who received linezolid in their BR were more likely to achieve SCC compared with those without linezolid (relative risk of 1.28; 95% confidence interval 0.99–1.6), although the time taken to achieve SCC was significantly longer in the linezolid group (mean time to SCC of 102.9 days for linezolid versus 65.4 days without linezolid). The effect of linezolid was modelled using the relative risk of SCC, which was assumed to represent the effect of linezolid on time to SCC.

Treatment discontinuation with a novel or Group 5 intervention was simulated in terms of both loss to follow-up and discontinuation of the intervention due to AEs.

The treatment algorithm for patients with MDR-TB – including drug treatment, dosages, and length-of-stay in isolated care – reflected recommendations made in local clinical guidelines and expert opinion [6–8, 25]. Surgery was excluded from this analysis as a treatment intervention, as guidelines recommend surgery in only a small minority of MDR-TB cases [6].

The health utility weights applied in the simulation model were adapted from a previous study in a low incidence setting [17], as local data for Germany are not available. Patients who occupied the active MDR-TB and lost to follow-up states were assigned the utility weight for active TB, while patients who occupied the sputum converted MDR-TB states were assigned a utility weight that was dependent on the time since conversion (a longer duration of sustained conversion was associated with an improvement in utility weight up to the utility weight for the general population [treatment completion]). Accordingly, the utility weight for sputum converted MDR-TB was estimated by linear interpolation of the weights for active TB (lower bound) and the general population (upper bound). All health utility weight inputs are summarized in Additional file 1: Table S1.

### Cost inputs

Direct medical costs were assumed to consist of drug acquisition costs, costs of treatment monitoring, costs of administered care (inpatient and outpatient care), costs of end of life care, and costs of managing AEs.

Unit costs for each drug in the BR, the cost of each monitoring resource, outpatient visits, and inpatient costs were sourced from publicly available tariffs and formularies in Germany [26]. Drug costs (€/2015) were based on the German Drug Directory (Lauer-Taxe Online 2015), and calculated based on the smallest pack available for the minimum period of treatment necessary, assuming use of generics when available [27]. The cost of resource use and monitoring was adapted from a previous study [11].

The cost of AEs included medication costs and monitoring costs, and was based on expert opinion and clinical study data [14] [analysis with data on file] [12, 28]. AEs that were at least potentially causally related to the investigational study drug and occurring in >5% of patients were included for each intervention. Inputs for the cost and duration of treatment-associated AEs are presented in Additional file 2: Table S2. The cost of electrocardiogram and liver enzyme monitoring was absorbed within overall monitoring costs. A summary of costs for the total costs of treatment, split by interventions and cost category (hospitalisations, outpatient costs, medication costs etc.) is displayed in Additional file 3: Table S3.

### Sensitivity analysis

Both probabilistic and deterministic sensitivity analyses were conducted following international recommendations [29]. In the deterministic simulations, key model parameters and assumptions were varied by ±20%, including clinical efficacy for each intervention, transition probabilities, utility weights, discount rates, the cost of AEs, and drug costs.

In probabilistic analyses, the likelihood of bedaquiline, linezolid, or delamanid plus BR being cost-effective versus BR alone was explored at different willingness-to-pay thresholds.

## Results

### Base-case results

Over the 10-year time horizon, the total discounted per-patient costs associated with the interventions under evaluation were €85,575 for bedaquiline, €81,079 for delamanid, €80,460 for linezolid plus BR, and €60,962 for BR only. Total costs were largely driven by TB drug costs and hospitalisation costs; costs associated with AE management were relatively small (Table 3).

**Table 3:** Per-patient and population level costs for the interventions included in the analysis

Patient-level							
Treatment strategy	Added costs for a novel or group 5 intervention (€)	Cost of BR treatment	Hospitalization costs (€)	Outpatient care (€)	Monitoring costs (€)	Adverse event costs of group 5 drugs (€)	Total (€)
Bedaquiline plus BR	30,799	28,652	24,038	98	1970	17	85,575
Delamanid plus BR	22,829	29,626	26,362	108	2152	2	81,079
Linezolid plus BR	20,302	29,968	27,392	107	2569	121	80,460
BR only	0	30,270	28,180	119	2393	–	60,962
Population-level, 100 patients with MDR-TB							
Bedaquiline plus BR	3,079,915	2,865,240	2,403,791	9833	197,014	1735	8,557,529
Delamanid plus BR	2,282,920	2,962,563	2,636,180	10,755	215,241	229	8,107,888
Linezolid plus BR	2,030,217	2,996,842	2,739,250	10,715	256,867	12,091	8,045,981
BR only	0	3,026,959	2,817,995	11,919	239,279	–	6,096,152

*AE* adverse event, *BR* background regimen, *TB* tuberculosis, *MDR-TB* multidrug-resistant tuberculosis

The differing cost of BR treatment across regimens displayed in Table 3 primarily reflects two model assumptions. Firstly, patients who fail to achieve sputum culture conversion in the first 20 months of treatment are considered treatment failures, and go on to receive further BR treatment in the secondary MDR-TB state until cure, loss to follow-up, or death. With add-on therapies, more patients achieve cure, fewer patients fail treatment, and fewer patients go on to receive BR in the secondary MDR-TB state. Secondly, the model assumes a difference in the mortality rate between culture-converted and unconverted patients. The higher rate of cure with add-on therapies means that patients will on average live longer, and consequently, a higher percentage of patients will complete the full course of BR treatment.

The total discounted cost and discounted QALYs for the single cohort of 100 patients assigned to bedaquiline plus BR was €8,557,529 and 479 QALYs, respectively (Table 4). The total discounted cost and discounted QALYs for the patients assigned to delamanid plus BR was €8,107,888 and 421 QALYs, respectively, and to linezolid plus BR, €8,045,981 and 391 QALYs, respectively. In terms of life-years gained (LYG), the total discounted LYG for the patients assigned to bedaquiline plus BR was 595; delamanid plus BR, 536; to linezolid plus BR, 507; and BR alone, 482 (Table 4).

**Table 4:** Incremental cost per QALY gained

Treatments ordered from least to most effective	Patient-level			Population-level (100 MDR patients)			Incremental cost per QALY gained (€)		
	Total cost, €	Total QALYs gained	Total LYG	Total cost, €	Total QALYs gained	Total LYG	Versus BR alone	Versus Linezolid plus BR	Versus delamanid plus BR
BR alone	60,962	3.68	4.82	6,096,152	368	482	–	87,484	38,703
Linezolid plus BR	80,460	3.91	5.07	8,045,981	391	507	87,484	–	2026
Delamanid plus BR	81,079	4.21	5.36	8,107,888	421	536	38,703	2026	–
Bedaquiline plus BR	85,575	4.79	5.95	8,557,529	479	595	22,238	5787	7774

*BR* Background Regimen, *QALY* Quality-Adjusted Life Year, *LYG* life-years gained

The incremental cost per QALY gained versus BR alone was €22,238 for bedaquiline, €38,703 for delamanid, and €87,484 for linezolid (Table 4).

**Fig. 2:**
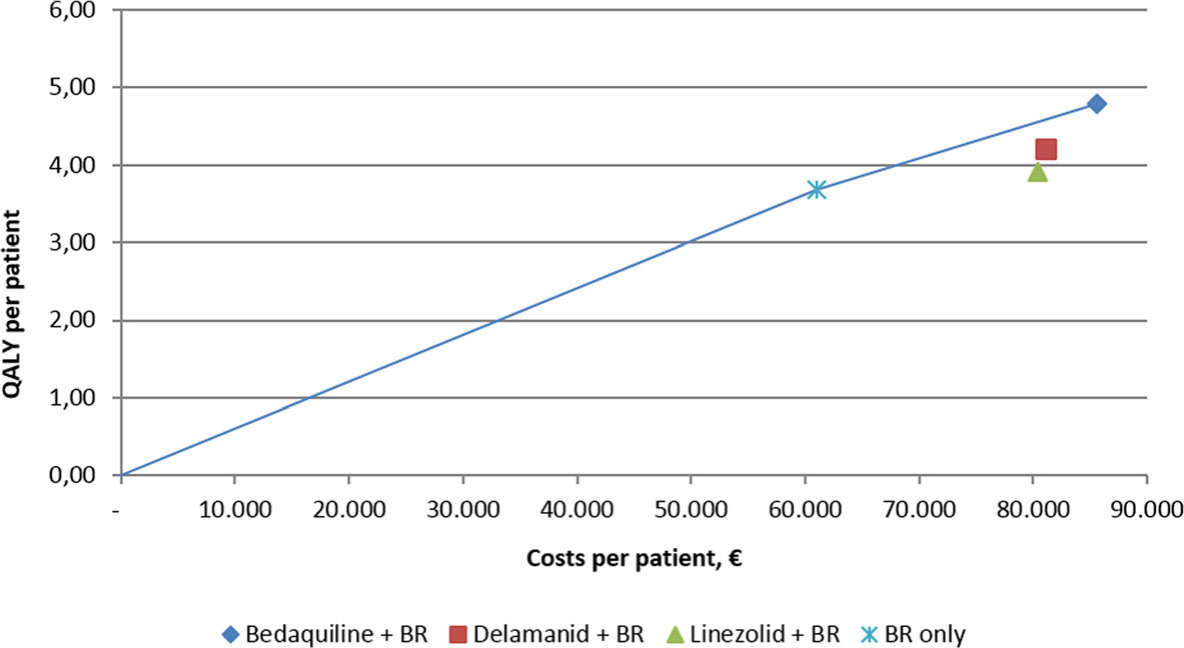
Cost-efficiency frontier. BR: Background Regimen; QALY: Quality-Adjusted Life Year

The results of the fully incremental analysis are presented graphically using the cost-efficiency frontier (Fig. 2), which compares the expected costs (x-axis) and benefits (y-axis) of each intervention in the evaluation [30]. The cost-efficiency frontier is plotted by connecting those treatments that represent the most cost-efficient use of healthcare resources, relative to any other therapy or their combinations.

In this analysis, BR alone and bedaquiline plus BR were considered the most cost-efficient interventions, with delamanid plus BR and linezolid plus BR being dominated by a combination of BR alone with or without bedaquiline. By excluding delamanid and linezolid from further consideration in the cost-efficiency frontier, the analysis shows that treatment with BR alone is the most cost-efficient therapy at willingness to pay thresholds up to €22,000 per QALY gained, with bedaquiline plus BR becoming the most cost-efficient therapy at thresholds greater than €22,000 (which falls below the informal threshold of €30,000 to €50,000 per QALY commonly applied in European healthcare systems).

### Sensitivity analyses

The results of the deterministic sensitivity analysis are presented in Table 5.

**Table 5:** One-way sensitivity analysis (per patient level)

Parameter	Variation	Cost / QALY				Incremental cost per QALY gained (€), Bedaquiline		
		BR alone	Linezolid plus BR	Delamanid plus BR	Bedaquiline plus BR	Versus BR alone	Versus Linezolid plus BR	Versus Delamanid plus BR
Base case		60,962 / 3.68	80,460 / 3.91	81,079 / 4.21	85,575 / 4.79	22,238	5787	7774
BR SCC rates at 6-months	+20% (37.2% SCC to 44.6%)	58,648 / 4.00	78,179 / 4.24	78,192 / 4.58	82,170 / 5.17	20,105	4316	6713
	−20%(37.2% SCC to 29.7%)	63,037 / 3.36	82,518 / 3.56	83,807 / 3.82	89,010 / 4.35	26,384	8196	9802
Effect of bedaquiline on SCC rates	+20%	60,962 / 3.68	80,460 / 3.91	81,079 / 4.21	84,384 / 5.01	17,711	3569	4162
	−20%	60,962 / 3.68	80,460 / 3.91	81,079 / 4.21	86,986 / 4.52	30,960	10,565	18,921
Maximum duration of linezolid treatment	42 to 24 weeks (duration of Bedaquiline treatment)	60,962 / 3.68	73,307 / 3.88	81,079 / 4.21	85,575 / 4.79	22,238	13,553	7774
Relapse after cure	+20%	61,232 / 3.65	80,732 / 3.87	81,421 / 4.16	85,716 / 4.77	21,809	5508	7092
	−20%	60,684 / 3.72	80,180 / 3.95	80,729 / 4.26	85,433 / 4.81	22,701	6094	8552
Utility weight for no cure	plus20%	60,962 / 3.95	80,460 / 4.16	81,079 / 4.46	85,575 / 5.02	22,988	5983	8056
	−20%	60,962 / 3.48	80,460 / 3.72	81,079 / 4.04	85,575 / 4.66	20,973	5465	7337
Utility for cure	Perfect health after cure	60,962 / 4.26	80,460 / 4.53	81,079 / 4.89	85,575 / 5.57	18,770	4905	6596
Discount rate	0% cost 3% outcomes	62,032 / 3.68	81,524 / 3.91	82,098 / 4.21	86,538 / 4.79	22,141	5672	7676
	6% costs 3% outcomes	60,039 / 3.68	79,547 / 3.91	80,210 / 4.21	84,765 / 4.79	22,340	5903	7875
	3% costs 0% outcomes	60,962 / 4.12	80,460 / 4.38	81,079 / 4.73	85,575 / 5.4	19,224	5015	6712
	3% costs 6% outcomes	60,962 / 3.33	80,460 / 3.52	81,079 / 3.79	85,575 / 4.29	25,473	6611	8914
Cost for BR medication	+20%	66,824 / 3.68	86,271 / 3.91	86,831 / 4.21	91,177 / 4.79	22,003	5550	7514
	−20%	55,099 / 3.68	74,649 / 3.91	75,327 / 4.21	79,974 / 4.79	22,474	6024	8034
Cost for AE’s	+20%	60,962 / 3.68	80,484 / 3.91	81,079 / 4.21	85,579 / 4.79	22,242	5764	7779
	−20%	60,962 / 3.68	80,436 / 3.91	81,078 / 4.21	85,572 / 4.79	22,235	5811	7768

*AE* Adverse event, *BR* Background regimen, *QALY* Quality-adjusted life-year, *SCC* Sputum culture conversion

In the one-way sensitivity analysis, the effect of bedaquiline on SCC was the most influential parameter, with ICERs of €17,711 (plus 20% more effective) and €30,960 (20% less effective) in comparisons with BR alone. Other influential parameters included the rate of SCC for BR alone (ICERs of between €20,150 [20% higher rate of SCC] and €26,384 [20% lower rate of SCC]), and the utility assigned to cured patients (ICER of €18,770 for perfect health post-cure versus €22,238 in the base case). The model results were not sensitive to assumptions on discounting rates, the cost of BR medication, the cost of AEs, or the rate of relapse after cure.

In comparisons of bedaquiline plus BR versus linezolid plus BR, all ICERs were below €15,000 per QALY gained. In the analysis where the duration of linezolid was reduced from 42 to 24 weeks (same duration as with bedaquiline plus BR), the ICER comparing bedaquiline plus BR versus linezolid plus BR increased from €5778 (base case) to €13,553 per QALY gained, which remains below a threshold of €30,000 per QALY, which is commonly applied informally in European healthcare systems. In comparisons of bedaquiline plus BR versus delamanid plus BR, all ICERs were below €20,000 per QALY gained.

**Fig. 3:**
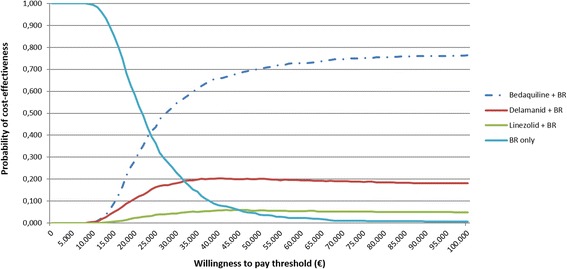
Multiway cost-effectiveness acceptability curve. BR: Background regimen

The results of the probabilistic sensitivity analysis are presented in Fig. 3. All data on probabilistic distributions, parameters and definitions used in the probabilistic sensitivity analysis are shown in Additional file 4: Table S4.

The probability that bedaquiline plus BR was the most cost-effective treatment strategy at a willingness to pay threshold of €30,000 was 54.5%, versus 22.9% for BR alone, 4.4% for linezolid plus BR and 18.2% for delamanid plus BR. At a higher threshold of €50,000 per QALY gained (informally, towards the upper limit of willingness-to-pay thresholds applied in European healthcare systems for orphan drugs), the probability that bedaquiline plus BR is a cost-effective alternative to existing interventions was greater than 70%.

## Discussion

The results of this study demonstrate that, in the German setting, adding bedaquiline, delamanid, or linezolid to a BR would lead to QALY and LY gains under a range of scenarios. The cost per QALY versus BR alone ranged from €22,238 for bedaquiline, to €38,703 for delamanid, and €87,484 for linezolid.

QALY gains for add-on therapies to a BR were driven by improved efficacy versus BR alone. As would be expected from adding interventions to a BR, higher total costs associated with add-on therapies were driven by increased drug acquisition costs, although total incremental costs were partially offset by reduced hospitalisation costs (resulting from improved sputum culture conversion compared with BR alone and therefore assuming less time spent in the inpatient setting). Monitoring costs and the costs of AE management had a relatively limited effect on the outcome, as the AEs were generally treatable with inexpensive medications.

Where multiple treatment options are available, it is important to identify which strategies provide the most efficient use of limited health care resources. This is achieved through fully incremental analysis where the cost-effectiveness of transitioning from one option to a more effective option is assessed. A key conclusion from this analysis was that a mix of strategies comprising BR alone and BR plus bedaquiline may yield greater QALY benefits than treatment with either linezolid or delamanid, but at higher total costs. More formally, linezolid and delamanid were dominated by a combination of BR alone and BR plus bedaquiline, and consequently, the administration of linezolid and delamanid would be considered a cost-inefficient use of available resources. Following standard practice [31], linezolid and delamanid were excluded from further cost-effectiveness analyses. The resulting analyses suggest that BR plus bedaquiline is the most cost-efficient treatment strategy at thresholds greater than €22,000 per QALY gained.

At the same time, the exclusion of linezolid and delamanid due to cost would not be clinically justifiable, given that dominance can only be achieved if some patients receive BR alone. Without access to novel therapies, some of these patients may go on to develop further drug resistance because of inadequate treatment with BR, possibly leading to active secondary MDR-TB with an excess morbidity and mortality burden. Moreover, there is a recognition that combining several new mechanisms of action will likely further enhance treatment outcomes [32]. Thus, MDR-TB treatment strategies should not be excluded based on dominance, given the severe consequences of poor outcomes in this population with a BR-only based treatment.

Previous studies have focused on the comparative effectiveness [33] or the cost-effectiveness of novel or Group 5 interventions compared with BR alone [15–18], but this is the first study to explicitly model cost-effectiveness across novel or Group 5 interventions for the treatment of MDR-TB within a consistent model structure. The results of this analysis are consistent with previous cost-effectiveness analyses of bedaquiline for MDR-TB [15, 17, 34], reinforcing that this intervention remains cost-effective under a range of assumptions and different country settings. The total costs for BR alone and ICER versus bedaquiline varied slightly to values reported in a previous cost-effectiveness analysis [34], due to a minor model variation in which treatment efficacy was linked to discontinuation over time: patients who leave the model (after failure to successfully treat active secondary MDR-TB) are assumed to derive no further benefit from treatment. Nevertheless, the conclusions are broadly consistent.

The results of the delamanid analysis presented here differ from a previous analysis by Diel and colleagues [18]. In the current analysis, delamanid plus BR was associated with an ICER of €38,703 per QALY versus BR alone, whereas in the Diel et al. publication, delamanid dominated over BR with an ICER of € -3494 per QALY gained. This discrepancy is very likely to reflect the different perspectives considered in these analyses: societal in the Diel analysis; payer in the current analysis, as well as the use of different data sources to inform the modelling by using patient-level data and the differing definitions of outcome parameters. To our knowledge, no economic evaluations of linezolid in MDR-TB have been published to date.

Due to the toxicity associated with MDR-TB therapies, it is important to consider the impact of AEs and their time of onset when comparing treatment options. This is the first study to comprehensively incorporate the costs of AEs associated with Group 5 interventions into a cost-effectiveness analysis of treatments for MDR-TB. It was not possible to assign utility weights to these AEs due to the lack of disease-specific data to inform utility loss associated with MDR-TB in the German setting. Although proxy data could – in theory – be substituted to inform utility loss, attempting to apply such data on top of a utility weight already heavily impacted by disutility due to BR side effects proved unfeasible in this analysis.

The development of utility weights for MDR-TB specific AEs would therefore be valuable to inform future cost-effectiveness analyses in MDR-TB. However, due to the comparative tolerability of the interventions considered in this analysis relative to other MDR-TB drugs, the conclusions are likely to hold even if such disutility were to be explicitly modelled. Alternatively, a quality-adjusted time without symptoms or toxicity (Q-TWIST) approach [35] could be considered. Although typically applied in oncology, such an analysis would capture disease- and treatment-related disutility, and would also allow the user to model the patient impact of the much-needed shortened treatment regimens that are currently in clinical trials.

Additional future modelling work may account for recent developments in the MDR-TB treatment landscape, looking at shorter simplified regimens or different combinations of new and current drugs. Trials are currently underway to assess the efficacy of innovative combinations of Group 5 or novel interventions, such as the 6-month combination regimen of bedaquiline, linezolid, and PA-824 being evaluated in the NiX-TB-(B-L-Pa) trial (NCT02333799) [36], as well as the STREAM study. The STREAM study is a Phase 3, multicentre, international randomised controlled trial aiming to assess the safety and efficacy of shorter MDR-TB regimens [37]. The second stage of the STREAM study will include two bedaquiline-containing arms [38], including an all-oral regimen. Further, the EndTB program aims to expand access to new TB drugs such as bedaquiline and delamanid in 16 countries [39], while an NIH-sponsored safety trial is planned in South Africa.

The analysis presented here was subject to a number of assumptions, which have been described, discussed, and justified in detail previously [17]. Briefly, the use of trial data for bedaquiline and delamanid may not accurately reflect results found in German clinical practice. However, the results were not found to differ substantially when interim data from real-world evidence studies from a European and African setting were applied in sensitivity analyses [40–42], and therefore, the results of this analysis can be assumed to be applicable in the real-world setting.

Secondly, the mortality imbalance observed in the bedaquiline C208 clinical trial was not captured here because no causal link to study medication was found. Further, newly published results of the C209 trial [43] as well as recent published interim data from the ongoing Compassionate Use program for bedaquiline in France, South Africa, and Latvia [40–42, 44], suggest a lower mortality rate than that observed in the C208 trial, justifying this assumption.

The third assumption relates to patients after leaving the model. Patients who were lost to follow-up were assumed to remain in this state until death and did not incur any costs, for the purpose of simplifying the model structure. Data are limited on the retreatment of such patients [45], and due consideration to treatment with third-line or even fourth-line regimens was beyond the scope of this analysis. Regardless, this assumption is unlikely to substantially impact the results of the analysis, given that the rate of loss to follow-up was assumed to be consistent between treatment strategies [17].

In addition, patients who achieved sputum culture conversion did not experience any lasting disutility and were assigned the same utility as patients in the general population, which is likely to be a simplistic assumption. However, data on the utility of patients who have been cured of MDR-TB are limited and in sensitivity analyses, the impact of varying utility weights for the ‘cured’ health state had a relatively small effect on the ICER.

Finally, heterogeneity between the studies considered in this analysis introduces an element of uncertainty, and can be considered a key limitation of the model structure. Relative efficacy outputs should be interpreted within the appropriate context, especially given that the hazard ratio of SCC was the most influential parameter in one-way sensitivity analyses.

Although the best available evidence was used at the time of model development (clinical trial data for bedaquiline and delamanid; German observational data for linezolid due to a lack of RCTs in MDR-TB), other studies exist that could be used to model relative efficacy. For example, clinical trials carried out in XDR-TB populations in the Chinese and Korean settings [46–49], respectively, demonstrated higher rates of sputum culture conversion for linezolid than were reported in the German study used in this analysis [13]. In addition, recent contributions to the literature reporting MDR-TB and XDR-TB outcomes for bedaquiline [43] and XDR-TB outcomes for delamanid [50] could also be considered in future research. Alternatively, the collection and application of direct comparative clinical trial data would allow for treatment effect to be modelled more objectively.

## Conclusions

The addition of bedaquiline, delamanid, or linezolid to a BR would result in QALY gains over BR alone when applied in the German healthcare system. Bedaquiline is likely to be the most cost-effective intervention for the treatment of MDR-TB, when added to a BR regimen at thresholds greater than €22,000 per QALY. These results may be used to inform MDR-TB treatment and reimbursement decisions within the German healthcare system and in other high-income countries.

## Additional files

Additional file 1: Table S1. Adverse events considered in the economic model [12, 14, 28, 51, 52]. (DOCX 22 kb)
Additional file 2: Table S2. Utility data used for health states in the economic model. (DOCX 13 kb)
Additional file 3: Table S3. Total costs of treatment in the MDR-TB cohort, split by intervention & cost category. (DOCX 13 kb)
Additional file 4: Table S4. Probabilistic distributions, parameters and definitions as used in the PSA. (DOCX 14 kb)

## Abbreviations

AE: Adverse event
BR: Background regimen
CI: Confidence interval
ICER: Incremental cost-effectiveness ratio
LYG: Life-year gained
MDR-TB: Multidrug-resistant tuberculosis
QALY: Quality-adjusted life-year
Q-TWIST: Quality-adjusted time without symptoms or toxicity
SCC: Sputum culture conversion
TB: Tuberculosis
XDR: Extensively drug-resistant
