# Supplementary material for: Cost-effectiveness of adding novel or group 5 interventions to a background regimen for the treatment of multidrug-resistant tuberculosis in Germany
Source: BMC Health Serv Res. 2017 Mar 8;17:182. doi: 10.1186/s12913-017-2118-2 (PMC5341441; doi:10.1186/s12913-017-2118-2)
Supplement: Additional file 1: Table S1. — Adverse events considered in the economic model [12, 14, 28, 51, 52]. (DOCX 22 kb) [file 12913_2017_2118_MOESM1_ESM.docx]

Table S1: Adverse events considered in the economic model

| **Adverse event** | **Frequency*, %** | | | **Medication** | **Unit cost,** € | **Median duration [51,52], days** | **Calculation** |
| --- | --- | --- | --- | --- | --- | --- | --- |
|  | **Delamanid [12]** | **Linezolid [28]** | **Bedaquiline [14]** |  |  |  |  |
| **Haematopoietic** | | | | | | |  |
| Anaemia | - | 27.4 | - | Blood transfusion | 320.00 | 2 | Expert opinion |
| Leukopenia | - | 7.3 | - | None – absorbed by monitoring | | - | - |
| Thrombocytopenia | - | 7.6 | - | None – absorbed by monitoring | | - | - |
| **Gastrointestinal** | | | | | | |  |
| Grouped (as reported) | - | 13.5 | - | As below | - | 7 | - |
| Nausea | - | - | 20.3 | Dimenhydrinate coated tablets or metoclopramide tablets | 23.91 | 7 | 400mg per day for 7 days; 50 mg per tablet = 8 tablets per day = 56 tablets; therefore, require 3 packs of 20 tablets at 7.97 EUR per pack |
| Vomiting | - | - | 16.5 |  | 24.84 | 7 | 30mg per day for 7 days; 10mg per tablet = 3 tablets per day = 21 tablets; therefore, 2x20 packs at 12.42 |
| Gastritis | - | - | 6.3 | Pantoprazole | 27.90 | 7 | 40mg per day for 7 days; 20-mg per tablet = 2 tablets per day = 14 tablets; therefore, 1x 14 pack at 13.95 |
| **Cardiovascular & respiratory** | | | | | | |  |
| Prolonged QT interval | 7.2 | - | - | None – already absorbed by monitoring costs | | - | - |
| **Nervous system, musculoskeletal, eye & psychiatric** | | | | | | |  |
| Headache | 2.5 | - | 6.3 | symptomatic NSAID, aspirin | 4.60 | 14 | 2000mg per day for 14 days; 500mg per tablet = 4 tablets per day = 56 tablets; therefore 2x30 tablet packs at 2.30 |
| Insomnia | 10.0 | - | 6.3 | Oxazepam or Zolpidem | 13.67 | 14 | 10mg per day for 14 days; 10mg per tablet = 1 tablet per day = 14 tablets; therefore 1x20 pack at 13.67 EUR |
| Arthralgia | - | - | 13.9 | symptomatic NSAID, Diclofenac | 16.43 | 16.5 | 75mg per day for 16.5 days; 25mg per tablet = 3 tablets per day = 49.5 tablets; therefore, 1x50 pack at 16.43 |
| Myalgia | - | - | 6.3 | symptomatic NSAID, aspirin | 4.60 | 14 | 2000mg per day for 14 days; 500mg per tablet = 4 tablets per day = 56 tablets; therefore 2x30 tablet packs at 2.30 |
| Dizziness | 3.4 | - | 6.3 | Dimenhydrinate coated tablets or metoclopramide tablets | 23.91 | 14 | 400mg per day for 7 days; 50 mg per tablet = 8 tablets per day = 56 tablets; therefore, require 3 packs of 20 tablets at 7.97 EUR per pack |
| Peripheral neuropathy | - | 30.9 | - | Amitriptyline, Gabapentin | 72.75 | 14 | 2400mg per day for 14 days; 300mg per tablet = 8 tablets per day = 112 tablets; therefore 3x50 packs at 24.25 |
| Optic neuritis | - | 8.0 | - | Prednisolone IV | 46.50 | 7 | 25mg per day for 7 days; 25mg per tablet = 1 tablet per day = 7 tablets; therefore 3x 3 pack at 15.55 |
| **Other** | | | | | | |  |
| Hyperuricaemia | - | - | 11.4 | Allopurinol | 12.36 | 7 | 300mg per day for 7 days; 300mg per tablet = 1 tablet per day = 7 tablets; therefore 1 x 30 pack at 12.36 |

IV: intravenous; NSAID: non-steroidal anti-inflammatory drug; QT: time between the start of the Q wave and the end of the T wave in the heart's electrical cycle

* AEs considered at least potentially causally related to study drug and occurring in >5% of patients were included.
