# Supplementary material for: Cost-effectiveness of adding novel or group 5 interventions to a background regimen for the treatment of multidrug-resistant tuberculosis in Germany
Source: BMC Health Serv Res. 2017 Mar 8;17:182. doi: 10.1186/s12913-017-2118-2 (PMC5341441; doi:10.1186/s12913-017-2118-2)
Supplement: Additional file 2: Table S2. — Utility data used for health states in the economic model. (DOCX 13 kb) [file 12913_2017_2118_MOESM2_ESM.docx]

Table S2: Utility data used for health states

| Health state | Utility value | Standard error | Reference |
| --- | --- | --- | --- |
| Patients with no cure | 0.68 | 0.14 | Jit, Stagg, Aldridge et al (2011) |
| Patients with treatment completed and cured (first year) | 0.79 | 0.04 | Jit, Stagg, Aldridge et al (2011) |
| Patients with treatment completed and cured (subsequent years) | 0.81 | 0.04 | Jit, Stagg, Aldridge et al (2011) |
| Patients lost to follow-up | 0.68 | 0.14 | Assumed to be the same utility weight as patients with active TB (no cure) |
| End of Life care | 0.68 | 0.14 | Assumed to be the same utility weight as patients with active TB (no cure) |
| Dead | 0.00 |  |  |
