# Supplementary material for: Cost-effectiveness of adding novel or group 5 interventions to a background regimen for the treatment of multidrug-resistant tuberculosis in Germany
Source: BMC Health Serv Res. 2017 Mar 8;17:182. doi: 10.1186/s12913-017-2118-2 (PMC5341441; doi:10.1186/s12913-017-2118-2)
Supplement: Additional file 3: Table S3. — Total costs of treatment in the MDR-TB cohort, split by intervention & cost category. (DOCX 13 kb) [file 12913_2017_2118_MOESM3_ESM.docx]

Table S3: Total costs of treatment in the MDR-TB cohort, split by intervention & cost category

| **Cost category** | MDR-TB cohort (n=100) | | | |
| --- | --- | --- | --- | --- |
|  | BR alone | Linezolid plus BR | Delamanid plus BR | Bedaquiline plus BR |
| Hospitalisation costs | € 2,817,995  [46.23%] | € 2,739,250  [34.04%] | € 2,636,180  [32.51%] | € 2,403,791  [28.09%] |
| Outpatient care | € 11,919  [<1%] | € 10,715  [<1%] | € 10,755  [<1%] | € 9,833  [<1%] |
| Anti-TB drugs (excluding group 5) | € 3,026,959  [49.65%] | € 2,996,842  [37.25%] | € 2,962,563  [36.54%] | € 2,865,240  [33.48%] |
| Anti-TB drugs  group 5 drugs |  | € 2,030,217  [25.23%] | € 2,282,920  [28.16%] | € 3,079,915  [35.99%] |
| Monitoring costs | € 239,279  [<1%] | € 256,867  [03.19%] | € 215,241  [<1%] | € 197,014  [<1%] |
| Treatment of adverse events costs |  | € 12,091 [0.15%] | € 229  [<1%] | € 1,735  [<1%] |
| Total costs | € 6,096,152 | € 8,045,981 | € 8,107,888 | € 8,557,529 |

Costs reported in € and are 2015 values. Values [in brackets] show the overall percentage the specific cost contributes towards the total costs.
BR: Background regimen; MDR-TB: Multidrug-resistant tuberculosis;
