# Supplementary material for: Cost-effectiveness of adding novel or group 5 interventions to a background regimen for the treatment of multidrug-resistant tuberculosis in Germany
Source: BMC Health Serv Res. 2017 Mar 8;17:182. doi: 10.1186/s12913-017-2118-2 (PMC5341441; doi:10.1186/s12913-017-2118-2)
Supplement: Additional file 4: Table S4. — Probabilistic distributions, parameters and definitions as used in the PSA. (DOCX 14 kb) [file 12913_2017_2118_MOESM4_ESM.docx]

Table S4: Probabilistic distributions, parameters and definitions as used in the PSA

Probabilistic distribution

| Health state | Transition to | Treatment | Distribution | Alpha | Beta |
| --- | --- | --- | --- | --- | --- |
| Active TB  (MDR-TB; secondary MDR-TB and XDR-TB) | Sputum culture converted | - | Multivariate normal (log-transformed variables) | See below | See below |
|  | Lost to follow-up | All arms | Beta – sampling range between 0 and 1 | 6.037 | 1560.457 |
|  | Death  MDR-TB no cure  MDR-TB cure | All arms | Beta – sampling range between 0 and 1 * | 16509  448.755 | 1333385  252086.258 |
| Lost to follow-up | Death | All arms | Beta – sampling range between 0 and 1 | 0.377 | 5.116 |
| Sputum culture converted | Active secondary MDR-TB (relapse) | All arms | Beta – sampling range between 0 and 1 | 7.838 | 788.289 |
|  | MDR-TB or active XDR-TB (relapse) |  | Beta – sampling range between 0 and 1 | 6.000 | 22.000 |
| Treatment completion and cured | Active secondary MDR-TB (reoccurrence) | All arms | Beta – sampling range between 0 and 1 | 15.991 | 7837.896 |
|  | MDR-TB or active XDR-TB (reoccurrence) | All arms | Beta – sampling range between 0 and 1 | 6.000 | 22.000 |
| Treatment withdrawal | | Sirturo | Beta – sampling range between 0 and 1 | 0.69 | 78.31 |
|  |  | Delaminid |  | 4.04 | 316.96 |
|  |  | Linezolid |  | 11.38 | 355.62 |
| Hazard ratio for sputum culture conversion | | Sirturo | Lognormal – positive and skewed distribution. Alpha and beta are on the log scale, and are sampled using the normal distribution and then transformed back to normal scale using the exponential function | 0.846 | 0.302 |
|  |  | Delaminid |  | 0.492 | 0.343 |
|  |  | Linezolid |  | 0.098 | 0.546 |

Note* Probability of death combined with the hazard ratio for death comparing MDR-TB versus TB alone. Hazard ratio is not sampled in probabilistic analysis.

| Covariate | Mean estimate (log-transformed) | Cholesky decomposition of covariance matrix | | |
| --- | --- | --- | --- | --- |
|  |  | Intercept | Trt | Scale |
| Weeks 0-8 | | | | |
| Intercept | 1.603 | 0.041 | 0.000 | 0.000 |
| Trt | -0.879 | -0.271 | 0.216 | 0.000 |
| Scale | -0.404 | 0.109 | 0.057 | 0.091 |
| Weeks 8-24 | | | | |
| Intercept | 1.731 | 0.070 | 0.000 | 0.000 |
| Trt | -0.180 | -0.319 | 0.315 | 0.000 |
| Scale | 0.609 | 0.079 | 0.041 | 0.106 |
| Weeks 24+ | | | | |
| Intercept | 2.106 | 0.048 | 0.000 | 0.000 |
| Trt | 0.588 | -0.152 | 0.164 | 0.000 |
| Scale | 0.939 | 0.056 | 0.026 | 0.076 |
